# Urethritis—Its Treatment

**Published:** 1876-03

**Authors:** Jno. Thad. Johnson

**Affiliations:** Professor of Anatomy and Lecturer on Venereal Diseases in the Atlanta Medical College


ATLANTA

MEDICAL AND SURGICAL JOURNAL.

VOL. XIII.] MARCH—1876. [No. 12.

### Original Communications.

V

### URETHRITIS- ITS TREATMENT.

BY JNO. THAD. JOHNSON, M.D.,
Professor of Anatomy and Lecturer on Venereal Diseases in the Atlanta Medical College.

*Lecture delivered before the Class.*

There are but few diseases we are required to treat oftener than this one. There are few for which more remedies have been proposed, or more plans been tried. It is a disease which at times yields readily, flattering the physician that he has at last hit upon the plan to relieve him of future annoying failures. I am not sure whether this occasional facility of cure depends most on the inherent nature of the particular case, or of the patient, or the source of contagion, or the exact and sometimes accidental adaptation of patient, time, and remedy. We all meet such cases; and on the other hand, we sometimes exert our utmost skill on a patient, and he a reasonably careful one, for weary weeks and months, without putting an end to his persistent discharge and eternal relapses. Very probably his next physician finds the same experience. The discharge has come to be the only evidence of disease—in fact, the symptom is practically the disease. These are the cases that rob the physician of his patience, and let the wind out of conceit engendered by some previous brilliant stroke. Such are the cases that send patients skipping from friend to friend, druggist to druggist, and doctor to doctor. Such the cases that pave the way for organic changes that creep insidiously for months and years, and at last do serious injury to the health, and even deprive its victim of his life. You will avoid much trouble by being always careful to cure your patient before he has reached this indefinite stage.

Suppose the patient presents himself in the early stage. If you could confine your patient to bed for several days, apply cold constantly to the offending member, internally and to the integument, often you would have no further trouble. But this is not a practicable plan. Your patient will not go to bed for a gonorrhœa; he will not "play sick" for an affection apparently so insignificant; and if you insist too much upon it, he will find a doctor with different views. He wants to remain at his employment, and thus prevent the discovery of his little indiscretion.

Shall we treat him locally or constitutionally, or combine the two? It is better to regard the affection as a tangible urethritis rather than a mysterious gonorrhœa; yet we find stages and symptoms benefited by the so-called constitutional specifics. The local treatment, however, is applicable in all stages; and so successful that very rarely do I exhibit any remedy whatever internally.

But to succeed in this way, you must know that your remedy is thoroughly applied. Your treatment is not really begun until your patient is perfect master of the little weapon I show you, called a syringe. Perhaps in years past he has perfected himself in the art; but let him demonstrate this fact before you; and if he is green in the calling, teach him; do not *tell* him how, but *show* him how. If you fail in this, he will fritter away precious hours and valuable days in sprinkling your injection over his linen, and over every portion of his person but his urethra. Provide him a proper instrument, always one of gutta-percha, if possible—but of whatever kind, see that the piston behaves perfectly, that it does not force the fluid at either end indifferently. There is no need for a long nozzle—the shorter the better. A short one causes less pain, and is inserted more easily. A novice with a sore urethra, a swollen prepuce, a slippery glans, a long pipe, and a loose piston, is but a burlesque on the graceful art of injection. Such a patient, without instruction, actually goes *untreated.*

You need not direct your patient to place one hand on the deep urethra. The fluid will not enter the bladder; and if it should, will do no harm, provided the injection be of proper strength. Your patient has need for both hands at the other extremity of the organ.

An apron of ordinary cotton goods, pinned to a band around the waist, will prevent the staining of your patient's linen more effectually than the bundle of rags you will so often find investing it.

You may choose from an endless list of remedies that have been applied by injection. These are generally of an astringent character. But do not fall into the habit of racing through the materia medica searching for the magic article that is to certainly make the unwelcome guest down at its bidding. You will not find it. It does not exist. Select a few, perhaps a half dozen—enough to play the changes on—that you believe to be the best; cultivate them, know them thoroughly that you may use them rationally. There is no necessity for complicated mixtures; they are too uncertain, notwithstanding they may sometimes answer admirably. A plain solution of your remedy in pure water, or mild medicated water, is best, and affords opportunity of regulating its strength.

There are a few maxims it would be well for you to remember.

You cannot "burn out" the disease. The idea is a pernicious one. Because a certain strength accomplishes so much, is no reason that twice the strength will confer twice the benefit. Some persons treat gonorrhœa as though aqua fortis were the natural food for a penis.

The urethra is as fastidious of its proper dose—much more so, indeed—than is the stomach.

Different strengths of the same remedy are really different remedies. Where a particular solution proves serviceable, one slightly weaker might prove inefficient, and one somewhat stronger might aggravate the trouble—hence the necessity of thorough knowledge of your weapons. A remedy too irritating will aggravate the disease—will even set up an inflammation in an innocent penis.

A very high authority, Dr. Van Buren, of New York, rightly says, that "the weakest injection that will do good is the proper strength."

The test of the proper strength is the pain produced by it; severe and long-continued pain indicates too great a strength; a very slight smarting, lasting a few moments, should be arrived at.

If the patient presents himself in the earlier stage, before the acute inflammation has run high, the disease may be often aborted, that is, it may never reach the inflammatory stage. A treatment called the abortive, formerly in vogue, now nearly obsolete, consisted in caustic injections of nitrate of silver—ten to forty grains to the ounce. This is extremely painful, dangerous, generally ineffectual, and is apt to prolong the disease if it does not chance to abort it. You can accomplish your object much more certainly, without pain and without danger, by the use of the same article; but in a manner I have termed the rational abortive treatment. I use it on a different principle, and in infinitely smaller doses. I do not cauterize the urethra with one injection, but make the application thoroughly and very frequently. That strength should be one-twelfth of a grain, applied thoroughly every two hours. One-eighth of a grain, every three or four hours will answer, but not so well in most persons. The former strength is preferable.

Many practitioners, accustomed to greater strengths, will ridicule this injection as mere child's play, I ask but a test of it, and will abide the result. Let nothing of the instructions I have laid down be omitted in this test. Unless the instructions are perfectly complied with, I am not responsible for the failure.

After a few applications the membrane becomes more sensitive to the remedy. What of discharge was present almost ceases. A flow of blood is often produced—resembling, in this respect, the caustic injections before alluded to.

When this effect is reached, lengthen the interval to six hours; or, what is a better plan, substitute sulphate of zinc (one or two grs. to ounce), acetate of lead (one gr. to ounce), or acetate of zinc (one-half gr. to the ounce). By this plan, carefully carried out, the discharge does not often become purulent, nor the inflammation advance. The disease, in short, is *aborted.* Your patient must continue the above injections (zinc, lead, etc.) for a number of days, lest the disease is again fanned into a vigorous flame, and your previous success be nullified.

There is another article that answers almost as well as the nitrate of silver, and, chemically, is less delicately hinged—that is, if distilled water is not accessible, that which is less pure will answer. I allude to sulphate of copper. One-fourth or one-half of a grain to the ounce, every two or four hours (two is better), is the proper application.

This plan, I repeat, is, above all others, the abortive treatment. If *properly carried out*, the disease, treated before the intense inflammation has supervened, will promptly subside in most instances. It is the treatment I rely upon implicitly. It has the further advantage of working no harm should the disease not be aborted—as it occasionally will not. In such case, it still constitutes a part of the systematic, rational treatment that is most efficient in a continued gonorrhœa.

If, despite this, the disease advances (as it occasionally will do) to the inflammatory stage, or has already reached it before the patient presents himself, you cannot succeed so promptly, but still may shorten the disease very much. The greater pathological changes that have arisen require some time for their resolution. The same remedies may be used, but with a different end in view. Nitrate of silver, or sulphate of copper, in the same strength but not so diligently applied, are also of service in this stage. More useful, perhaps, are the acetate of lead, acetate of zinc, sulphate of zinc, in the strengths above indicated, Tannin (five grs. to the ounce), alum (five to ten grs. to the ounce), are useful, but not generally so efficient as the articles already mentioned. Remember, it is not the long list of remedies to be used, but the thorough understanding of a few, that will render your treatment satisfactory to both physician and victim. Keep the discharge, and with it its concomitant condition, well in subjection. The inflammation is the cause of the discharge, but I am inclined to think the discharge, in turn, aggravates the disease. In this inflammatory stage, acetate or bi-carbonate of potassa, with an occasional saline laxative, may prove of service, though I rarely find it necessary to resort to them.

In what is known as the third stage, the acute inflammation—and with it the pain—subsides. It now behooves you to be earnest in your treatment, or your enemy may flourish indefinitely. In this stage your patient's habits may conflict with your treatment. The pain has departed, and there is no obstacle but his self-control to the exercise of his organ.

I may here digress to speak of the so-called specifics. They are not useful in the first stage, nor in the earlier period of the second. They are of service in the later second, and in the earlier portion of the third stage. When the inflammation first creeps deep into the urethra, in the neighborhood of the bladder, then you may, by their use, emphasize your local treatment.

There is a symptom attending this stage, in some cases, that calls for these remedies. I allude to the frequent and urgent desire for micturition. The neck of the bladder may become too irritable, and the smallest quantity of urine, the mere fancy or a thought of the patient, excites the most intense desire for emptying the bladder; the patient must beat a retreat wherever he may be, and stand not upon the order of his going. The same symptom may be met in other affections of the urinary tract. You may control this symptom and benefit the disease itself at this stage by the exhibition of oil of yellow sandal wood, copaiba, or cubebs. I value them in the order mentioned. They should be pushed until the desired effect is produced, or other effects arise that demand their discontinuance. I do not commence with less than a half dram of the first two, and increase rapidly. The oil of flea-bane (erigeron canadensis) has had the same virtues attributed to it, but my experience with it is limited. These remedies may be given in emulsion, or in capsule, to disguise their taste. Unpleasant irritations often attend their use. Their odor may proclaim the patient's misfortunes to his friends. It is in but the small minority of cases that I find it necessary to resort to these remedies.

In this, the third stage, I make use locally of the remedies above suggested for the earlier periods. The strength must be very slightly increased. Frequent changes of the remedy must be made, if the duration of the disease require it. This substitution may be made with advantage every three or five days. The remedy that I find of great service at this date, as an injection, is the muriated tincture of iron (twelve to twenty-five minims to the ounce of water). Its constringing properties seem to restore tone to the mucous membrane. I have found no remedy more useful. I have not given it trial in the earlier stages.

It is in this stage, as I have intimated, that we may find the disease obstinate. This is especially true of neglected cases; hence the greater importance of a prompt cure in the earlier period. Give every attention to the patient's habits. He should not exercise the organ, either by coition, masturbation, or rough manipulation in search of discharge. Such indiscretions will nullify any amount of treatment. Nothing is more important than attention to this point.

When the disease has at last disappeared, the patient must continue two or three daily injections for some time. For, without such attention, you will often find it more difficult to *keep* him cured than to cure him.

I repeat it: everything depends upon continuing treatment for *many* days after the discharge has totally disappeared; for if it return, then the trouble begins. There is then a condition engrafted that knows no tendency to cure; a habit is contracted that the penis has not energy to throw off. It is neglect of this precaution that allows so much chronic gonorrhœa. It is this neglect that has engendered among some patients a contempt for a doctor's treatment of gonorrhœa. It is this that sends such ones racing to druggists and quacks; that sends them to some more experienced friend, whose pockets bristle with infallible prescriptions, and who is ever ready to bombard the unfortunate penis on the slightest provocation. Your patients will object to continuing treatment after they consider themselves relieved, but you must force them to it by warning them of the consequences of failure to comply with your instructions.

A few words with regard to diet: my instructions during treatment are: "Pursue your ordinary diet." If the inflammation runs high, the febrile reaction will partially destroy the appetite; hence injunction to that effect is unnecessary. I am aware my instructions are at variance with the teachings of many authorities, but reasoning, and attentive observation in many cases, render me positive in my convictions. If the patient has been accustomed to the use of stimulants, I do not deprive him of them entirely. I regard wine as most injurious of all such beverages. I have not been able to persuade myself that gin possesses any curative power in gonorrhœa, though you will find such an idea prevalent.

So far, as you have seen, I have not alluded to any distinction between specific urethritis (gonorrhœa) and simple urethritis. The symptoms are the same. It is said that simple urethritis is altogether milder, and yields more readily to treatment. Simple urethritis, unconnected with surgical procedures, is a rare, a very rare, disease. It may occur. It does occur—sometimes. Your patients, those who have some special motive for concealment, will often affirm by all manner of asseveration that they are innocent of any possible specific origin. You need not dispute them, but go on with your treatment. Treat them as though they had acquired their ailment "in the good old way"—as most often they have.

Before leaving this subject, I must speak of gonorrhœa in the negro race; for I find it necessary to pursue a plan differing from that already given you. This necessity arises from the negro's carelessness and ignorance. Rarely can we teach him the proper use of the syringe; could we do so the same plan of treatment would answer at least as well. This class of patients never present themselves until the disease has advanced a number of days. We are compelled to resort to copaiba or cubebs, or similar remedy, and they answer the purpose very well. I am inclined to believe that the disease is less obstinate in the negro; certainly, he is less careful of a perfect cure.

A few words regarding a condition called gleet, characterized by a discharge very slight, almost colorless, and discoverable only after the urethra has remained undisturbed by urine for some time, as in the morning. While this discharge, lasting for but a short time, may be regarded as the natural diminution, or method of cure, of the urethritis—a prolongation, indeed, of the third stage, and yielding to its treatment—yet it sometimes grows to be a most persistent affection (or symptom).

Although a strictly gleety discharge is not contagious, it may be easily fanned into one that is at once purulent and contagious, constituting the "bastard gonorrhœa" of recent writers. Undue exercise of the penis will again and again convert this innocuous scant discharge into a profuse and purulent one; and every such exacerbation renders the final cure more and more difficult.

General congestion of the urethra, or patches of such congestion, or incipient stricture, are the more frequent causes of this discharge. When this gleet has existed for a long while, refusing to yield to treatment, you should introduce a full-sized smooth metallic sound, of size to moderately distend the urethra. You do this first as a diagnostic test. If you detect a stricture, cure it; the gleet is but a small matter compared with the stricture; and disappears by treatment of its cause. There may be a slight deposit of the stricture material, too slight and too immature to obstruct your instrument, but yet sufficient to *parent* the gleet.

If, in such chronic cases, your instrument finds no stricture, still it is the best remedial agent at your service. Introduce it again in four or six days; continue this, and your patient will be cured. It is rarely necessary or useful to spread a medicated ointment on the bougie; the cold steel answers. Its pressure and distension prove tonic to the tissues, relieve congestion, perhaps cure a slight ulceration, and arrest an inappreciable stricture. It is a remedy too much neglected, but one that will prove of great service if you use it properly. While pursuing this plan you may resort to tonic remedies, and those with special tendency to the mucous membranes. Tincture of iron and cantharides are of some service. In the intervals of the introduction of the bougie, injections of sulphate of copper, or tincture of iron, as above directed, will prove of very great value.

